# Unveiling the Enigma of Multiple Odontomas in Pediatric Dentistry: A Rare Clinical Presentation

**DOI:** 10.7759/cureus.62654

**Published:** 2024-06-18

**Authors:** Mridula Goswami, Mona Pattu, Smriti Johar, Vashi Narula

**Affiliations:** 1 Pediatric and Preventive Dentistry, Maulana Azad Institute of Dental Sciences, New Delhi, IND

**Keywords:** benign, children, dental management, conservative surgical management, odontoma

## Abstract

Odontomas are the most common tooth-like hamartomatous odontogenic tumours, which are usually asymptomatic and found on routine radiographs. Odontomas are commonly classified into two types: complex and compound odontomas. The most common consequences of odontoma are tooth impaction and/or delayed tooth eruption. The present report describes a unique case of multiple complex odontoma in the anterior maxillary region in a 12-year-old young patient, which is a rare occurrence. The uniqueness of this case report is in the location, number, and complexity of the complex tumours. Early diagnosis, proper treatment planning, appropriate management, and regular follow-ups of these odontomas helped in complete removal and prevented recurrence even after 12 months of follow-up.

## Introduction

Paul Broca coined the term "odontoma" in 1867, originally referring to any tumour or tumour-like lesion of odontogenic origin [1]. Odontomas are developmental anomalies caused by the excessive proliferation of fully differentiated epithelial and mesenchymal cells, which give rise to functional ameloblast and odontoblast. They can be an unintended discovery during a routine radiological examination and are typically linked to difficulties in tooth eruption [2]. Odontomas are considered the most common type of odontogenic tumour or growth, which can manifest in various forms at different locations within the oral and maxillofacial region [3]. The incidence of odontogenic tumour ranges from 0.002% to 0.1%, with odontomas being the most prevalent (20-67%) [4]. Regezi et al. examined a sample of 706 jaw tumours and reported that odontomas constituted 67% of the sample [5].

The exact aetiopathogenesis of odontomas is not known, but several factors have been associated with its development. Researchers report that a combination of local, developmental, and genetic factors play a role in the development of odontomas. Local factors include trauma, inflammatory reactions, and persistent infections; hereditary factors include syndromes like Gardner's syndrome, Hermann's syndrome, familial colonic adenomatosis, and basal cell nevus syndrome; and developmental factors involve odontoblastic hyperactivity or persistence of a portion of the dental lamina (cell rests of Serre) [6].

Odontomas commonly occur in permanent dentition and are rarely reported in association with primary teeth. They are usually asymptomatic, are slow growing, may or may not be causing bony expansion, and are frequently discovered during routine radiography [7]. In spite of their benign nature, odontomas can cause various dental problems such as delayed eruption of adjacent teeth, pain, swelling, infections, adjacent tooth resorption, and rarely cyst formation. Therefore, they require careful diagnosis and treatment [8,9].

The most used classification given by WHO in 2005 divided odontomas into two types: (a) compound odontomas and (b) complex odontomas [10]. Tooth-like structures known as denticles in compound odontomas often resemble miniature teeth that may be arranged in clusters or irregular patterns. Complex odontomas are composed of disorganized dental tissues in irregular clusters of masses which include a mixture of enamel, dentin, cementum, and pulp tissue. The incidence of compound odontomas ranges between 9% and 37% and that of complex odontomas is between 5% and 30% [11]. Compound odontomas are more prevalent in the anterior segment of the jaws (61%), while complex odontomas are more common in the posterior segment (59%) [12]. No gender predilection is present for both types of odontomas [13].

Odontomas are most commonly diagnosed in the first two decades of life; thus, the pediatric dentist plays an important role in the diagnosis and management of odontomas. Also, pediatric dentists work to prevent any complications like malocclusion and promote growth and development by addressing odontomas early on.

The treatment options for odontomas vary according to the size and location of the tumour. Nowadays, the concept of odontoma as a benign tumour has evolved into a hamartomatous malformation. A hamartoma is a benign, local malformation consisting of an abnormal combination of cells and tissue. An odontoma is considered a hamartoma because it is a benign tumour-like growth composed of normal dental tissues that have grown in a disorganized manner [14]. Odontomas are well-capsulated lesions so they can be managed using conservative surgical excision which includes the selective removal of denticles with a focus on enucleation of connective tissue capsule.

The present case report entails the surgical management of a rare case of multiple complex odontomas in a 12-year-old girl. In this pediatric case, the sheer number of odontomas extracted, numbering 12, makes this case unique.

## Case presentation

Key findings

Clinical findings are as follows: (a) age being 12 years but missing left maxillary central and lateral incisor (21,22) and (b) absence of any pain or swelling.

Radiographic findings are as follows: (a) large radiopaque calcified mass in the region of 21,22 which seemed to be obstructing the eruption of permanent teeth and (b) impacted left maxillary central and lateral incisor (21,22).

**Figure 1:**
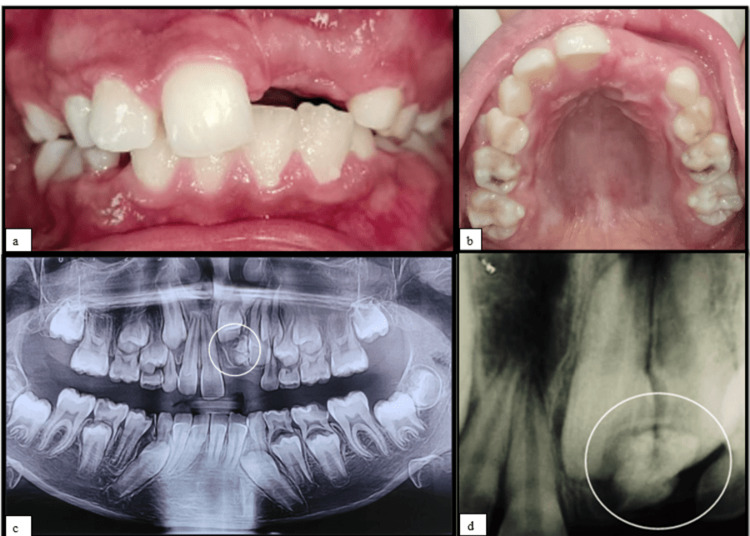
(a, b) Intraoral preoperative pictures showing missing left maxillary permanent central incisor. (c) Orthopantomogram showing large radiopaque calcified mass wrt 21,22. (d) Intraoral periapical radiograph showing large radiopaque mass with radiodensity as that of a tooth wrt impacted 21,22 21: left maxillary permanent central incisor; 22: left maxillary permanent lateral incisor; wrt: with respect to

A 12-year-old girl visited the Department of Pediatric and Preventive Dentistry, with a chief complaint of missing teeth in the upper left front tooth region for the past four years for which the parents and the patient were aesthetically concerned. The patient had no history of pain and swelling. Her medical history was non-contributory with a negative family history for hypodontia or hyperdontia. Intraoral examination showed a missing maxillary left central incisor (21) (Figure 1a, 1b). Radiographic investigations, intraoral periapical radiograph (IOPA), and orthopantomogram (OPG) were advised. The OPG and IOPA showed an irregular large radiopaque calcified mass surrounded by areas of radiolucency related to impacted 21,22 with an approximate length of 3-4 cm away from the alveolar crest region (Figure 1c, 1d).

Surgical procedure

**Figure 2:**
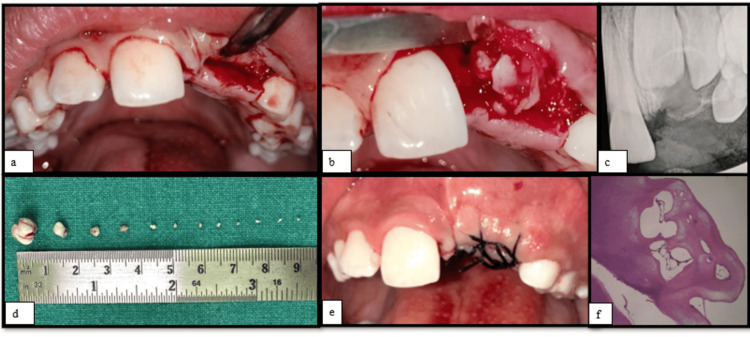
(a) Intraoperative view showing crestal incision in the region of 21 and crevicular incision wrt 11 and 22. (b) Intraoperative view showing surgical exposure of the calcified mass. (c) Postoperative RVG. (d) Multiple calcified masses extracted from surgical site measuring in varying sizes. (e) Postoperative view showing sutures. (f) Histopathological slide showing conglomerated mass dentin along with loose fibrous connective tissue RVG: radiovisiography; 11: right maxillary permanent central incisor; 21: left maxillary permanent central incisor; 22: left maxillary permanent lateral incisor; wrt: with respect to

There are many methods of removal of odontoma. Surgical removal of odontoma is the most common and conventional method. Alternate and emerging methods of removal include using lasers and piezoelectric and endoscopic approaches. In this case, the conventional surgical method of removal was followed. Under aseptic precaution and local anaesthesia, a crestal incision was given in the centre of the alveolar region, in the region of 21, and a crevicular incision was placed in the region of 11 and 22 (Figure 2a). A full labial periosteal flap was raised. After flap elevation and exposing the surgical site in the region of 21, a large mass of calcified structure (Figure 2b) was removed using a periosteal elevator without damage to adjacent teeth. An intraoperative radiograph was taken for confirmation. It was found that a few more calcified smaller masses were present in the same region which were previously hidden by the large mass. After adequate bone removal, one by one smaller masses were luxated and extracted under the guidance of digital radiography (Figure 2d). The surgical site was irrigated with Betadine, and the flap was approximated and sutured with 3/0 silk (Figure 2e) after achieving haemostasis. Antibiotic, anti-inflammatory, and pain relief coverage were prescribed, and the patient was kept on regular follow-ups.

Postoperative care

The patient was prescribed antibiotics, anti-inflammatory, and pain relief coverage for five days. The patient was instructed to apply cold compresses externally 3-4 times for the first 24 hours to reduce swelling and inflammation. To maintain adequate oral hygiene, the patient was advised to gently brush with a soft-bristled toothbrush, avoid the surgical site, and use an antiseptic mouthwash or saltwater rinse to keep the area clean, especially after meals. A cold soft diet was recommended for 24 hours. The patient was asked to continue taking a soft diet for one week post-surgery, including avoiding hard, crunchy, or spicy foods. The patient was advised to drink plenty of fluids. The parent was educated on signs of infection such as increased pain, swelling, redness, or discharge from the surgical site and was asked to report to the department in case of any such issue. The patient complied with all the instructions reinforced repeatedly on every follow-up appointment.

Follow-up appointments

The patient was kept on long-term follow-up as it is crucial to ensure optimal outcomes, particularly in pediatric cases. The patient was recalled after 24 hours, one week, one month, six months, eight months, and one year.

24 Hours Follow-Up

After 24 hours, the surgical site was assessed for signs of infection such as pain, abnormal swelling, bleeding, and tissue response. Postoperative oral hygiene was evaluated, and instructions were reinforced to the patient.

One Week Follow-Up

After a week, the patient was called for suture removal. Suture removal was done along with Betadine irrigation of the surgical site. The patient's postoperative period was uneventful with satisfactory healing. A follow-up radiovisiography (RVG) revealed no abnormal radiological findings (Figure 2c).

One Month Follow-Up

Reassessment of the surgical site was done. Overall healing of the surgical site and surrounding tissues was satisfactory. Follow-up RVG did not reveal any abnormal bone radiolucency that may indicate any signs of infection.

Eight Months Follow-Up

**Figure 3:**
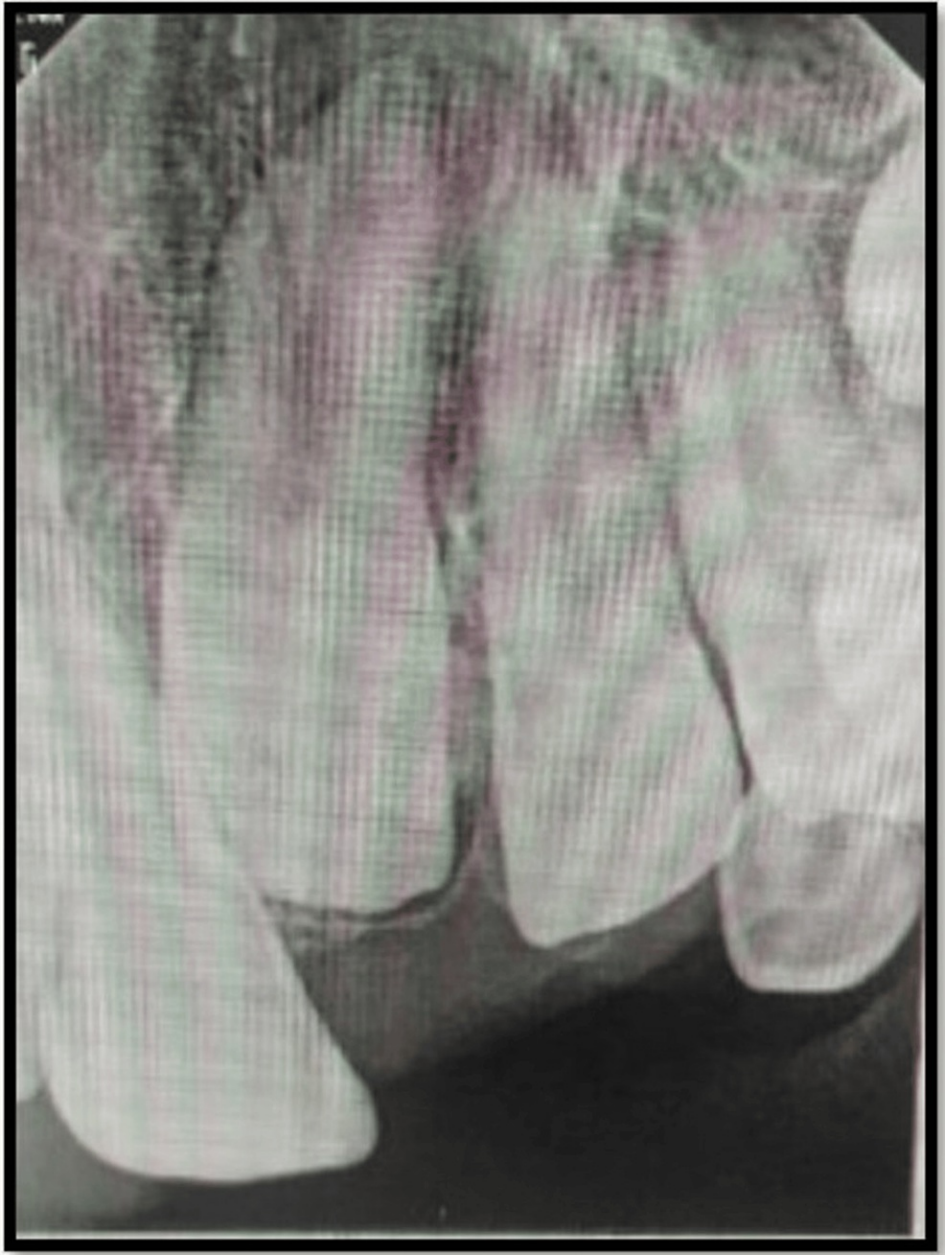
Eight-month follow-up RVG RVG: radiovisiography

The 8-month follow-up RVG showed root development (Nolla stage 9) wrt permanent maxillary central and lateral incisors (Figure 3).

Eleven Months Follow-Up

**Figure 4:**
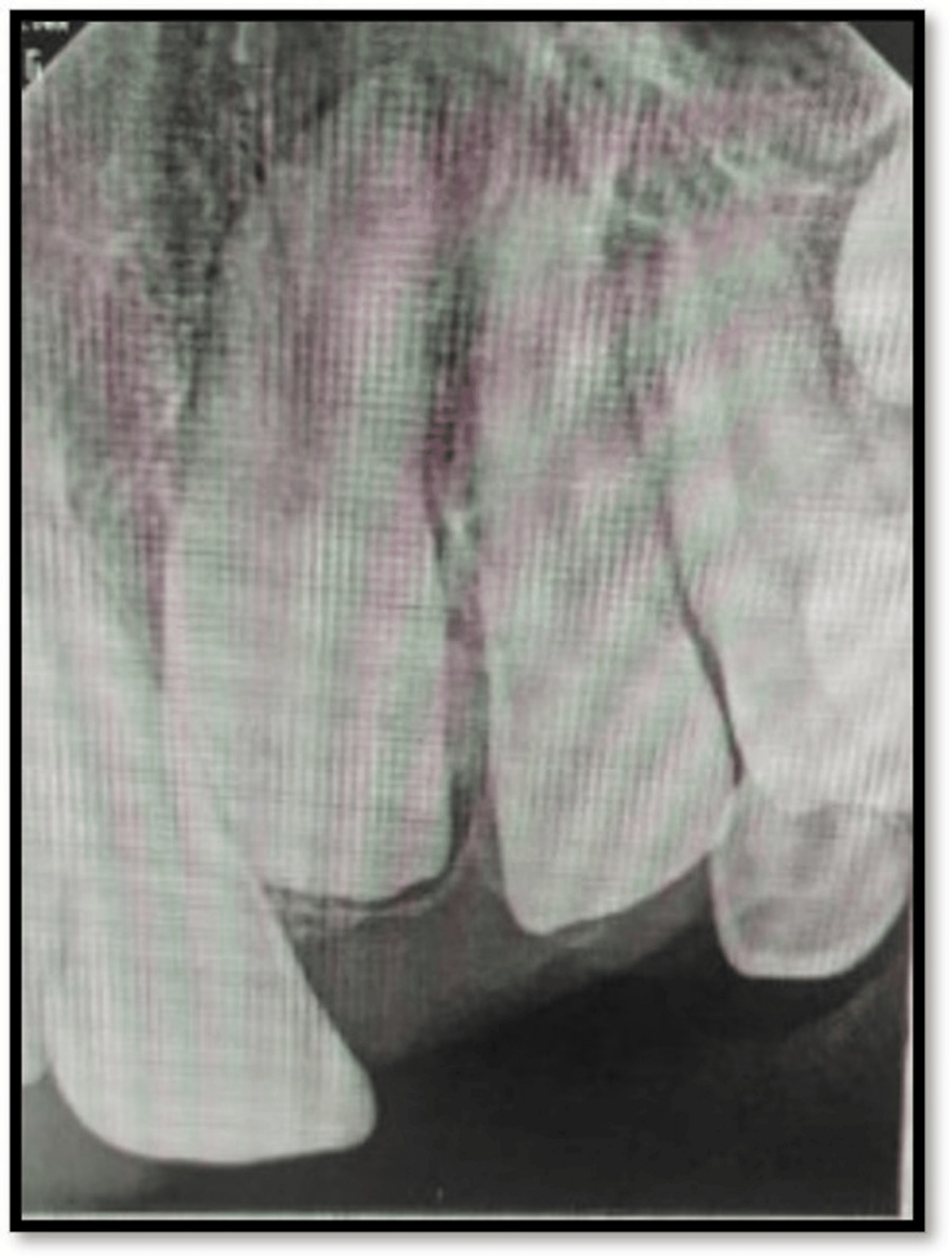
Eleven-month follow-up RVG RVG: radiovisiography

The 11-month follow-up RVG showed root development (Nolla stage 10) wrt permanent maxillary central and lateral incisors (Figure 4).

Histopathological findings

The extracted specimen was stored in formalin and transported for histopathological examination. The slide staining was done using eosin and haematoxylin. On histopathological examination, the decalcified hard tissue masses revealed conglomerate masses of tubular dentin exhibiting a prominent predentin layer with associated odontoblasts. Focal areas of pulp tissue were observed as loose fibrous connective tissue with plump fibroblasts and varying-sized blood vessels in the centre of dentinal tissue. Areas showing periodontal ligament were noticed at the periphery of the cemental tissue. Focal areas representing enamel spaces were also observed. An impression of complex composite odontomas was provided (Figure 2f).

## Discussion

Odontoma is a relatively common hamartomatous malformation found in the oral and maxillofacial region. The diagnosis of odontoma can be made at any age, although it is more common in the first two decades of life. In the scientific literature, three clinical presentations of odontoma have been identified, intraosseous, extraosseous, and erupted, with intraosseous odontomas being the most common [15]. Table 1 shows the classifications of odontomas.

**Table 1:** Classification of odontomas

Year	Author	Types
1914 [16]	Gabell et al.	1. Epithelial
		2. Composite (epithelial and mesodermal)
		3. Connective tissue
1937 [17]	Worth	1. Ectodermal origin (enameloma)
		2. Mesodermal origin (dentinoma, cementoma)
		3. Mixed ectodermal and mesodermal origin (complex composite odontomas, compound composite odontomas, geminated odontomas, dilated odontomas, including dens in dente)
1946 [18]	Thoma and Goldman	1. Geminated composite odontomas
		2. Compound composite odontomas
		3. Complex composite odontomas
		4. Dilated odontomas
		5. Cystic odontomas
1952 [19]	Robinson	Tumours originating from epithelial and mesenchymal dental forming tissues
2005 [20]	Barnes et al.	1. Complex odontomas
		2. Compound odontomas

Complex odontoma is most commonly found in the posterior mandibular region; however, in this case, it was located in the maxillary anterior region. Also, multiple odontomas have been rarely reported in young children. In this present case, multiple radiopaque calcified structures with a radiolucent (dark) halo surrounding the mass were found on radiographs with no resemblance to the dental structure, and clinically, after removing one large odontoma, sequential RVGs revealed the presence of additional small radiopaque calcified masses, which were later removed one by one [21].

In one other case reported by Shetty et al. in 2013 [22], surgical management of multiple complex odontomas was done to facilitate the eruption of the maxillary right permanent incisor in a 12-year-old female patient.

Various differential diagnoses for complex odontoma include ameloblastic fibroma, cementoblastoma, and supernumerary teeth [23]. Ameloblastic fibroma is a benign odontogenic tumour which may present as a mixed radiopaque-radiolucent lesion resembling an odontoma. Cementoblastoma is typically associated with the roots of the involved teeth. Irregular-shaped supernumerary teeth can also be considered as a mimic for odontomas. However, odontomas are highly radiopaque, coaggregate masses which can sometimes resemble enamel and dentin; thus, they should be differentiated from other conditions for proper diagnosis, treatment planning, and risk assessment.

Pediatric dentists are often the first to diagnose odontomas in children during routine dental examinations or when parents report dental concerns for missing or unerupted teeth. It is critical that pediatric dentists who care for children understand the wide range of differential diagnoses and with skills for surgical management considerations for odontomas. Regular follow-up appointments are essential for assessing the child's progress and addressing any residual issues.

Future complications

This patient has completed 11 months of follow-up and is free from any complications and unfavourable outcomes in both clinical and radiographs. While the removal of an odontoma in a young patient usually results in positive outcomes, there are potential future complications that can arise. Being aware of these possibilities allows for proactive monitoring and management. Pre- and postoperative complications of the surgery can be excessive bleeding, postoperative infection, hematoma formation, strictures, fibrosis, and dehiscence of bone. Removing an odontoma may delay the eruption of adjacent or overlying teeth, leading to impaction of teeth or teeth eruption in an abnormal position. Surgical removal of an odontoma can sometimes lead to root resorption or damage to adjacent teeth. The development of roots in permanent teeth may be disrupted, leading to shorter or malformed roots. In some cases, teeth adjacent to the surgical site may become ankylosed (fused to the bone), leading to impaction of associated permanent teeth and alignment issues of adjacent teeth. Bone resorption can also be a complication after odontoma removal.

Long-term outcomes

Successful removal of the odontoma often allows previously impacted teeth to erupt normally. With proper follow-up care, teeth can erupt in their correct positions with proper alignment, reducing the need for extensive orthodontic treatment later. Odontomas typically have a low recurrence rate after complete surgical removal [24]. However, long-term follow-up and monitoring help in early detection if any recurrence occurs, ensuring timely intervention.

Uniqueness of this case

There was the presence of multiple complex odontomas occurring in the anterior region of a young pediatric patient. A large single odontoma was identified on radiographic examination, but on clinical exploration during surgery, multiple smaller odontomas were found, a total of 12 in number in one single region, and this finding is rare.

## Conclusions

Multiple odontomas can cause discomfort and may be associated with various clinical manifestations, depending on their size, location, and impact on surrounding structures. The early identification and treatment of odontomas are crucial as they can help to prevent the occurrence of complications, preserve oral function, and address aesthetic concerns. Complete surgical removal of odontomas guided by digital radiography is the best treatment modality to avoid recurrence. Care must be taken to completely assess the surgical site for any residual masses during the surgical procedure to ensure complete excision of tumour.
